# Chitosan/Bioactive Glass Microparticles Enriched with Therapeutic Metal Ions for Bone Tissue Engineering

**DOI:** 10.1021/acsabm.5c00930

**Published:** 2025-07-29

**Authors:** Leonard Bauer, Zoya Hadzhieva, Iva Bazina, Meng Li, Faina Bider, Lucija Vlahović, Hana Kaňková, Aldo R. Boccaccini, Anamarija Rogina

**Affiliations:** † 112589 University of Zagreb Faculty of Chemical Engineering and Technology, Trg Marka Marulića 19, HR-10001 Zagreb, Croatia; ‡ Institute of Biomaterials, University of Erlangen-Nuremberg, Cauerstraße 6, 91058 Erlangen, Germany; § FunGlass - Centre for Functional and Surface Functionalized Glass, Alexander Dubček University of Trenčín, Študentská 2, 911 50 Trenčín, Slovakia

**Keywords:** bioactive glass, chitosan, microparticles, therapeutic metal ions, cytotoxicity, ion release

## Abstract

The application of divalent bioactive metal ions, such as Cu^2+^, Zn^2+^, and Mn^2+^, emerges as a growth factor-free approach for bone defect regeneration. Delivery of those ions can be achieved by organic or inorganic phases through desirable rapid or sustainable release in order to stimulate specific cell responses. In this work, bioactive ions were incorporated into both phases, chitosan (Cht), via chelation reactions, and mesoporous bioactive glass nanoparticles (MBGNs), by doping. The BG/Cht composites with undoped and Cu-, Zn-, or Mn-doped MBGNs were produced as spherical microparticles with a narrow size distribution and an average size of 42–45 μm via electrohydrodynamic atomization. Swelling studies showed enhanced water uptake in the complete cell culture medium with values between 2.5 and 3.1 compared to phosphate-buffered saline (2.2–2.5). Ion release experiments in phosphate-buffered saline revealed a pronounced release of silicon and calcium up to 7 days for all samples. A sustained release of manganese ions from the MnBG/Cht sample was detected for up to 14 days. A precipitated layer of calcium phosphates on all composites, except on the MnBG/Cht samples, confirmed the materials’ bioactivity after 21 days in simulated body fluid. Indirect cytotoxicity tests indicated that the materials were generally nontoxic to human osteosarcoma (MG-63) cells at concentrations below 1 mg/mL. However, direct contact assays with MG-63 and human dermal fibroblast (HDFa) cells revealed concentration-dependent cytotoxic effects, particularly for MnBG/Cht microparticles at a concentration of 0.5 mg/mL. Vascular endothelial growth factor (VEGF) expression analysis on MG-63 and HDFa cells demonstrated that only a higher concentration of MnBG/Cht significantly enhanced the angiogenic response in MG-63 cells, likely due to the decreased cell viability and oxidative stress generated by the redox activity of Mn^2+^ ions. Our results show that composite microparticles have good potential in the design of microparticulate systems with tailored properties through the combination of bioactive metal ions.

## Graphical abstract

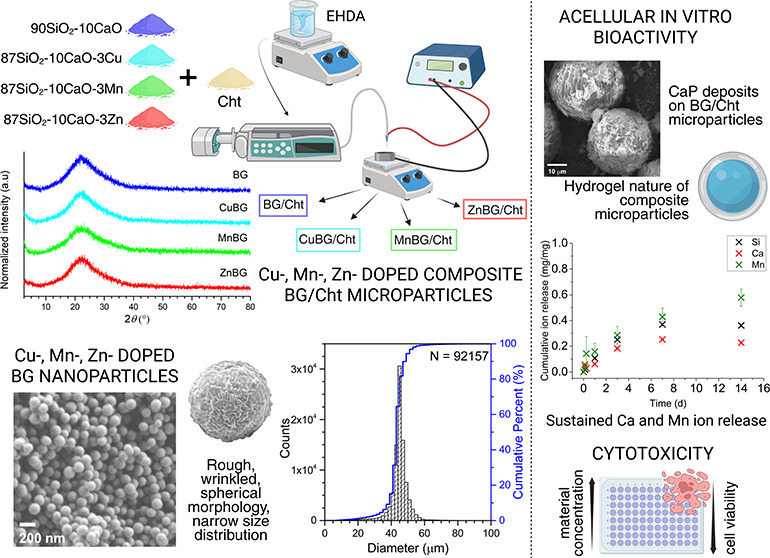

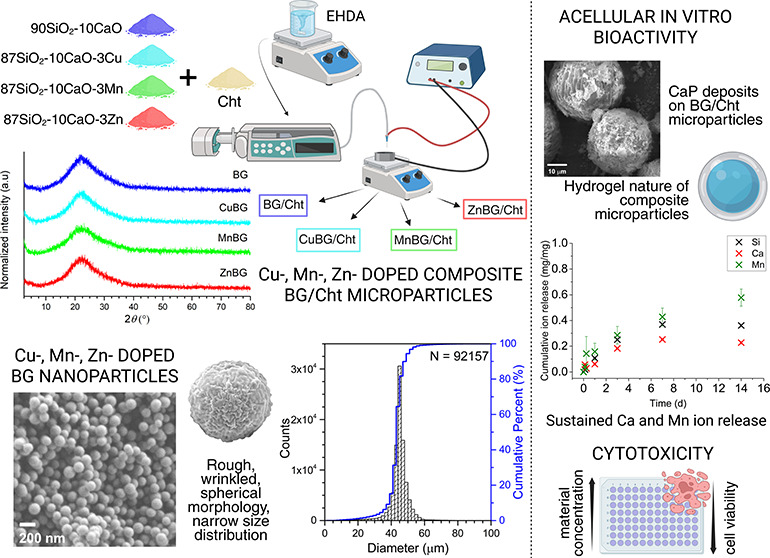

## 1 Introduction

In bone tissue engineering (BTE), organic–inorganic materials are extensively used since they have demonstrated superior properties with respect to their individual application.^1^ Inorganic fillers such as calcium phosphates (CaPs) and bioactive glasses (BGs) are mainly investigated as bioactive, osteoconductive, and osteoinductive components for composite materials.^2,3^ The ability to form strong bonds with bone tissue and promote new bond and blood vessel formation makes them highly interesting bioactive components for BTE.^4^ Since bone naturally serves as a reservoir for various ions involved in metabolic processes, ion-doped bioceramics and BGs can be designed to closely mimic the composition of native bone tissue.

When incorporated into BGs, metal ions such as magnesium, strontium, copper, and zinc can improve osteoblast activity, antibacterial properties, and bone formation *in vitro* and *in vivo*.^5,6^ The influence of therapeutic ions on osteogenesis and angiogenesis has recently been of great interest.^7^ For instance, as an important intracellular cation and a cofactor of many enzymatic reactions, magnesium released from SiO_2_–CaO–Na_2_O–P_2_O_5_–K_2_O–MgO bioactive glass supported mesenchymal stem cell (MSC) viability and induced osteogenic differentiation.^8^ Strontium- and zinc-doped BG enhanced bone formation by stimulating osteoblast activity and inhibiting bone resorption.^9^ Besides the therapeutic ions mentioned, increasing attention has also been directed toward copper and manganese ions.

Copper ions are involved in the activity of several transcription factors (via hypoxia-inducible factor 1 (HIF-1) and proline hydroxylase) and have been shown to stimulate endothelial cell proliferation and enhance angiogenesis *in vitro*.^10^ Furthermore, copper-doping improved angiogenic and osteostimulatory properties of bioactive glass scaffolds.^11^ Mn^2+^ ions increase the ligand-binding affinity of integrins, which mediate cellular interactions with the extracellular matrix (ECM) and activate cell adhesion.^12^ Manganese ions showed increased pro-osteogenic properties when doped in mesoporous BG nanoparticles (MBGNs); however, the positive osteogenic stimulation was accompanied by a cytotoxic effect in a dose-dependent manner.^13^

In the past few decades, polymeric microparticles have gained more attention in tissue engineering, not only as drug delivery carriers but also as microcarriers for large-scale expansion and differentiation of adherent cells, reinforcement in bioinks for 3D bioprinting, platforms for the development of tissue and disease models, and injectable systems tailored to defect sites.^14^ In contrast to 3D scaffolds, microparticles serving as hosts and carriers for cells in an injectable system could provide a bottom-up approach to tissue engineering. High surface-to-volume ratio and ability to mimic nanostructure and biochemical cues of native extracellular matrix make the microparticulate system an effective strategy for tissue regeneration.^15^

Chitosan-based materials have been broadly investigated in tissue engineering as they mimic the extracellular matrix, providing an optimal microenvironment for cell adhesion, proliferation, and differentiation.^16^ The stability of microsized chitosan-based materials is usually improved by chemical reactions involving cross-linkers, such as genipin or glutaraldehyde, leading to lower dissolution of material caused by the polycationic nature of chitosan.^17,18^ Recently, physical cross-linking by the formation of the metal-ion chitosan complex has been proposed,^18–22^ which can be a less toxic alternative to chemical cross-linkers. Successful production of chitosan-based microparticles (microgels) via the electrohydrodynamic atomization (EHDA) process has been accomplished at different quantities of Cu^2+^ ions.^23^ Copper ions stand out as suitable cross-linkers for chitosan chains where amino and hydroxyl groups participate in strong complexation, resulting in stable chitosan microparticles even at slightly acidic conditions. The stability of chitosan-copper microparticles under enzymatic degradation can be modulated by the concentration of Cu^2+^ ions involved in the complexation reactions.^23^ Nevertheless, careful optimization of the physical and biological properties is paramount for copper-containing biomedical materials.

In the present study, bioactive composite microparticles were produced as potential carriers of biologically active ions by combining both components, organic and inorganic. Sol–gel derived MBGNs were doped with Cu^2+^, Mn^2+^, and Zn^2+^ and could be released in a particular manner influencing targeted cell responses, such as osteogenesis and angiogenesis.^13,24,25^ MBGNs possess a larger specific area and porosity and induce CaP deposition in simulated body fluid (SBF).^26^ As the organic phase, chitosan enriched with Cu^2+^ ions through complexation reactions was used; this could provide a quick release of Cu^2+^ in physiological medium.^27,28^ The incorporation of bioactive ions into organic and inorganic phases can achieve rapid and sustained release of therapeutic ions, thus influencing the biological properties of materials. To the best of our knowledge, this is the first time a chitosan-copper matrix and ion-doped MBGNs were processed into spherical microparticles that could be used as individual microparticulate systems carrying specific bioactive ions or as a combination of microparticulate systems with tailored biological properties. Composite microparticles produced by an electrodynamic atomization process possess uniform and narrow size distribution, good bioactivity after 21 days in SBF, and sustained ion release in phosphate-buffered saline (PBS) for 14 days. Another innovative aspect of the composites presented in this work is the ability to decouple the ion release kinetics of ions that are released from the MBGNs from that of ions being released directly from the chitosan matrix. This approach offers the possibility of designing the release profiles of different ions (or even the same ion, e.g., Cu in the present case), tailoring them to the target application. This possibility is not available for MBGN incorporation in pure (non-ion-loaded) chitosan matrices, which constitute the majority of previous work in the literature.

## 2 Materials and Methods

### 2.1 Synthesis of Undoped and Cu-/Mn-/Zn-Doped Mesoporous BG Nanoparticles

Mesoporous bioactive glass nanoparticles of the nominal composition (mol %) 90SiO_2_-10CaO, 87SiO_2_-10CaO-3ZnO, 87SiO_2_-10CaO-3MnO, and 87SiO_2_-10CaO-3CuO were synthesized by the sol–gel method as reported previously.^29^ Briefly, 24 mL of tetraethyl orthosilicate (TEOS, 98%, Sigma-Aldrich, Germany) and 96 mL of ethanol (96% VWR, Austria) were added under magnetic stirring to a solution composed of 36 mL of ammonium hydroxide solution (28%, VWR, Austria), 200 mL of distilled water, and 64 mL of ethanol. After 30 min, 2.9 g of calcium nitrate tetrahydrate (99%, Sigma-Aldrich) and varying amounts (corresponding to the nominal compositions of BGNs) of Zn­(NO_3_)_2_·6H_2_O (Sigma-Aldrich, Germany), Mn­(NO_3_)_2_·4H_2_O (Sigma-Aldrich, Germany), or Cu­(NO_3_)_2_·5H_2_O (Sigma-Aldrich, Germany) were added and stirred for 1.5 h to obtain 87SiO_2_-10CaO-3ZnO, 87SiO_2_-10CaO-3MnO, and 87SiO_2_-10CaO-3CuO, respectively. The obtained nanoparticles were collected by centrifugation and washed two times with deionized water and ethanol, respectively, before drying at 60 °C overnight. Finally, the dried samples were calcinated at a heating rate of 2 °C/min up to 700 °C with a 2 h dwell time.

### 2.2 Production of Composite BG/Chitosan Microparticles

Chitosan (Cht)-copper­(II) ion solution as a polymer matrix was prepared as described in previous studies.^23,30^ Briefly, 1.0 wt % chitosan solution was prepared by dissolving chitosan powder (Chitoscience chitosan 85/100, Heppe Medical Chitosan GmbH, Germany) in 1% solution of acetic acid for 2 h at ambient conditions. Then, chitosan–copper­(II) ion complex solution was prepared by mixing the appropriate volume of copper acetate monohydrate solution (VWR International BDH, Belgium) into the specific volume of chitosan solution followed by stirring for 2 h. The molar ratio of chitosan’s amino groups and cupric ions was 1:0.0915 (the approximate amount of Cu^2+^ ion in the final solution is ∼3 wt %).

The BG/Cht suspensions were prepared by homogenizing the appropriate weight of BG nanoparticles with respect to the chitosan weight (*w*(Cht)/*w*(BG) = 90/10). The prepared systems were denoted according to the type of dopant (Cu, Mn, or Zn): CuBG/Cht, MnBG/Cht, and ZnBG/Cht, respectively, and BG/Cht for the composite with undoped BG.

The electrohydrodynamic atomization (EDHA) process was conducted as follows: a syringe (Becton Dickinson, France) was filled with the prepared composite suspension (10 mL), and processing parameters were set as summarized in Table 1. A saturated solution of magnesium nitrate (Mg­(NO_3_)_2_·6H_2_O, Sigma-Aldrich, Germany) was used to avoid fluctuations in relative humidity during the EHDA process. The needle was positively charged, and the collector was grounded. 50 mL of 5 wt % NaOH (p.a., Lach-Ner, Czech Republic) solution (gelation medium) was used as the collector. The required voltage for the EHDA process was determined according to the formation of a stable Taylor cone-jet mode. After that, a gelation medium with formed microgels was stored in falcon tubes for the next 2 h. Microgels were then washed with demineralized water until pH neutral and left in the water for 2 h. Thereafter, microgels were dehydrated with 96% ethanol (Gram-Mol, Croatia) for 1.5 h, dried with acetone (acetone exchanged three times; Lach-Ner, Czech Republic), and left under ambient conditions for solvent evaporation.

**Table 1:** Experimental Conditions of the EHDA Process

Parameter		Unit	Value
Dimensions of the chamber (L/W/H)		cm	30/30/25
Flow rate of the solution		mL/h	5
Needle gauge		G	25
Distance between the needle tip and the collector		cm	10
Concentration of NaOH		wt %	5
Volume of NaOH		mL	50
Temperature of the chamber		°C	27 ± 1
Relative humidity in the chamber		%	67 ± 4
Applied voltage	BG/Cht	kV	15 ± 1
	CuBG/Cht		14 ± 1
	ZnBG/Cht		15 ± 1
	MnBG/Cht		14 ± 1

### 2.3 Characterization of Mesoporous BG Nanoparticles and BG/Chitosan Microparticles

#### 2.3.1 Morphological and Structural Characterization of Undoped and Cu-/Mn-/Zn-Doped BG Nanoparticles

The particle morphology was analyzed by scanning electron microscopy (SEM, Auriga CrossBeam, Carl Zeiss Microscopy GmbH, Germany). The particles were dispersed in ethanol and placed on conductive aluminum tape without sputter coating. SEM images were taken at an accelerating voltage of 1 kV.

An energy-dispersive X-ray (EDX) spectrometer (X-MaxN Oxford Instruments, United Kingdom) coupled with SEM was used to confirm the chemical composition of the particles. EDX data was collected at an electron-accelerating voltage of 15 kV and a working distance of 6 mm.

Fourier transform infrared (FTIR) spectroscopy was conducted using an IRAffinity-1S spectrophotometer (SHIMADZU, Japan) in transmission mode. The spectra were recorded in the wavenumber range of 4000 to 400 cm^–1^ with a resolution of 4 cm^–1^ and a scan speed of 2.3 scans per minute.

X-ray diffraction (XRD) analysis was performed by using a MiniFlex 600 diffractometer (Rigaku, USA) with Cu Kα radiation. The diffraction patterns were collected in the 2θ range of 0° to 80° with a step size of 0.020° and a scan rate of 2° per minute. Before analysis, the samples were dispersed on low-background silicon wafers (Bruker, AXS).

#### 2.3.2 Size Distribution and Morphology of BG/Chitosan Microparticles

The size distribution of BG/chitosan microparticles was estimated using an inverted fluorescence microscope (Olympus IX3 equipped with a Hamamatsu ORCA-Flash 4.0 camera and CellSens image analysis software), while the morphology was imaged using SEM (Tescan Vega III Easyprobe, Czech Republic). Before SEM imaging, the samples were sputter-coated with gold–palladium for 45 s.

The cross-section of the composite microparticles was analyzed by SEM-EDX. To obtain the cross-section, the samples were embedded in paraffin and cut into slides with 5 μm thickness using a microtome (Accu-Cut SRM 200 Rotary, Japan).

#### 2.3.3 Swelling Behavior of BG/Chitosan Microparticles

The swelling behavior of BG/chitosan microparticles was investigated by immersion in phosphate-buffered saline (PBS) and Dulbecco’s Modified Eagle’s Medium (DMEM; w: 4.5 g/L glucose; w: 4 mM l-glutamine; w: 1.5 g/L NaHCO_3_; w: 1.0 mM sodium pyruvate; Cytion, Germany) supplemented with 10% fetal bovine serum (FBS) (Gibco, Thermo Fisher Scientific, MA, USA) and 1% antibiotic solution (penicillin/streptomycin, P/S; 10.000 units/mL penicillin, 10 mg/mL streptomycin; Sigma-Aldrich, Merck KGaA, Germany) at room temperature for 24 h. The swelling was estimated as the volume ratio of microparticles obtained after immersion in solutions and dry microparticles, *V*_wet_/*V*_dry_ (μm^3^/μm^3^). The dry and swollen samples were imaged by a BA200 binocular microscope (light microscope; Motic Instruments, Spain) with Motic Images Plus 2.0 software. Figures were processed by ImageJ 1.53e software,^31^ assuming the microparticle sphericity of 1.

#### 2.3.4 Ion Release

The ion release behavior of the composite microparticles was evaluated by immersing 10 mg of each composition in 20 mL of PBS (pH 7.4) and incubating the samples at 37 °C with shaking at 90 rpm for up to 14 days. To prepare the PBS solution, PBS pellets were placed in distilled water, stirred at room temperature until they were fully dissolved, and stored in a clean, dry container. Each sample composition was tested in triplicate, and additional control tubes with 20 mL of PBS were prepared to serve as blank groups.

At predetermined time points (0.125, 0.250, 1, 3, 7, and 14 days), 10 mL of supernatant was collected from each sample after centrifugation, and the volume was replenished with fresh PBS. The collected supernatants were aliquoted and stored at 4 °C until further analysis. This procedure was repeated at each time point.

To ensure the accuracy of the elemental analysis by ICP-OES, the sampled solutions were acidified to pH 2 after sample collection by adding 20 μL of concentrated HNO_3_ (conc. 67–69%, Analytika Ltd., Czech Republic). This acidification step is performed to avoid changes in ion concentrations in collected samples, particularly precipitation/complexation processes that may occur in saturated testing media during the time between sampling and ICP analysis. The ionic concentration in the supernatants was then measured using inductively coupled plasma optical emission spectroscopy (ICP-OES; Agilent 5100 SVDV, Agilent Technologies, Inc.) to determine the release profiles over the 14 days. Results are expressed as cumulative release with respect to the nominal composition of undoped and doped MBGN.

#### 2.3.5 In Vitro Bioactivity of BG/Chitosan Microparticles

The bioactivity test was performed in simulated body fluid, 27 mM HCO_3_-Tris-SBF. The SBF solution was prepared according to the recipe of Kokubo et al.^32^ The mass-to-volume ratio of the microparticles and SBF solution was set at 1 mg/mL. The samples were incubated at 37 °C for 21 days under static conditions. The SBF solution was replaced by a fresh solution every third day. After incubation, the microparticles were washed with demineralized water and left to dry for 7 days at 37 °C. The inorganic deposits were analyzed by SEM-EDX with an electron beam energy of 10 keV. Before imaging, the samples were sputtered with gold and palladium for 45 s.

#### 2.3.6 Indirect Cytotoxicity Assay

The indirect cytotoxicity of composite microparticles was evaluated by treating human osteosarcoma (MG-63) cells (product no. 86051601-1VL, Sigma-Aldrich, Germany) with extracts from the samples at different material concentrations (0.01, 0.1, and 1 mg/mL). UV-sterilized samples were incubated in DMEM (Gibco, Germany), supplemented with 10 vol % fetal bovine serum (FBS, Sigma-Aldrich, Germany) and 1 vol % penicillin/streptomycin (Pen-Strep; Sigma-Aldrich, Germany), at 37 °C with 5% CO_2_ for 1 and 7 days. After incubation, supernatants were collected by centrifugation for 10 min, followed by filtration and storage at 4 °C.

For cytotoxicity testing, MG-63 cells were seeded into 24-well plates at a cell density of 1 × 10^5^ cells per well and incubated for 24 h. After incubation, the medium was removed, and the cells were fed with the sample extracts and incubated for an additional 24 h. 1 vol % WST-8 solution (Sigma-Aldrich, Germany) was then added to each well for colorimetric analysis of cell viability. After a 4 h incubation, the absorbance was measured at 450 nm using a 96-well plate reader. The viability of the treated cells was calculated with respect to the nontreated ones.

#### 2.3.7 Direct Cytotoxicity Assay

A direct cytotoxicity test was performed on human dermal fibroblasts (HDFa; Gibco, Thermo Fisher Scientific, MA, USA) and MG-63 cells (Cytion, Germany) using the MTT (3-(4,5-dimethylthiazol-2-yl)-2,5-diphenyl-2*H*-tetrazolium bromide; Sigma-Aldrich, Merck KGaA, Germany) assay. The cells were seeded in 96-well plates at a density of 5000 cells per well in 200 μL of a complete cell culture medium. MG-63 cells were cultured in DMEM, while HDFa cells were cultured in EMEM basic medium (MEM Eagle, w: 2 mM l-glutamine; w: 1.5 g/L NaHCO_3_; w: EBSS; w: 1 mM sodium pyruvate; w: NEAA; 820100c Cytion, Germany). Both media were supplemented with 10% FBS and a 1% antibiotic solution. Cells were incubated for 24 h in a humidified incubator at 37 °C and 5% CO_2_ to adhere. Following adhesion, the cells were treated with the material at different concentrations (0.1 and 0.5 mg/mL) and incubated for 24, 48, and 72 h. After incubation, the medium containing the material was removed, and the cells were rinsed in PBS (Gibco, Thermo Fisher Scientific, USA). Then, 40 μL of MTT solution (0.5 mg/mL, diluted in complete culture medium) was added, followed by a 4 h incubation. Subsequently, 170 μL of DMSO (Gram-Mol, Croatia) was added to dissolve formazan crystals, and the samples were incubated for 20 min with constant shaking. The absorbance was measured at 560 nm by using a plate reader (Glomax-Multi, Promega, WI, USA). Cell viability was determined as a percentage relative to that of untreated control cells.

#### 2.3.8 Quantification of Vascular Endothelial Growth Factor (VEGF) by ELISA

The quantification of VEGF in the supernatant was done according to the manufacturer’s protocol using the Human VEGF SimpleStep ELISA Kit (Abcam, UK) after 72 h of HDFa and MG-63 cell culture in direct contact with the materials. The protein concentration in the cell supernatants was determined using a calibration curve generated with the absorbance value of standards with known VEGF concentrations (800, 400, 200, 100, 50, 25, and 12.5 pg/mL), and the absorbance of the tested samples was measured at a wavelength of 450 nm.

### 2.4 Statistical Analysis

The results are presented as mean values ± the standard deviation. To determine statistical differences, a two-way analysis of variance (ANOVA) was applied, followed by a Tukey’s *post hoc* test for group comparisons. Statistically significant differences between groups are marked with an asterisk (*).

## 3 Results

### 3.1 Characterization of Undoped and Cu-/Mn-/Zn-Doped Mesoporous BG Nanoparticles

SEM analysis revealed the formation of spherical nanoparticles with estimated sizes of up to 200 nm, while EDX spectra indicated the presence of Si, Ca, and dopants corresponding to the metal-doped BG composition, as seen from Figure 1. The incorporation of each dopant did not cause any significant differences in morphology compared to the undoped BG nanoparticles. The FTIR spectra of undoped and ion-doped MBGNs exhibited bands at 1049–1055 cm^–1^, 794–804 cm^–1^, and 443–445 cm^–1^ associated with Si–O–Si stretching, bending, and rocking vibrations, respectively.^33^ The amorphous nature of the BG nanoparticles was indicated by a broad band at around 2θ 23°, showing no detectable crystalline phase after calcination at 700 °C. This could imply the incorporation of CuO, MnO, or ZnO into the amorphous phase of BG nanoparticles. STEM analysis of similar undoped and doped MBGNs prepared by the same synthesis protocol^34^ showed well-defined mesopores with uniform distribution, which is also expected for the MBGNs in this work.

**Figure 1:**
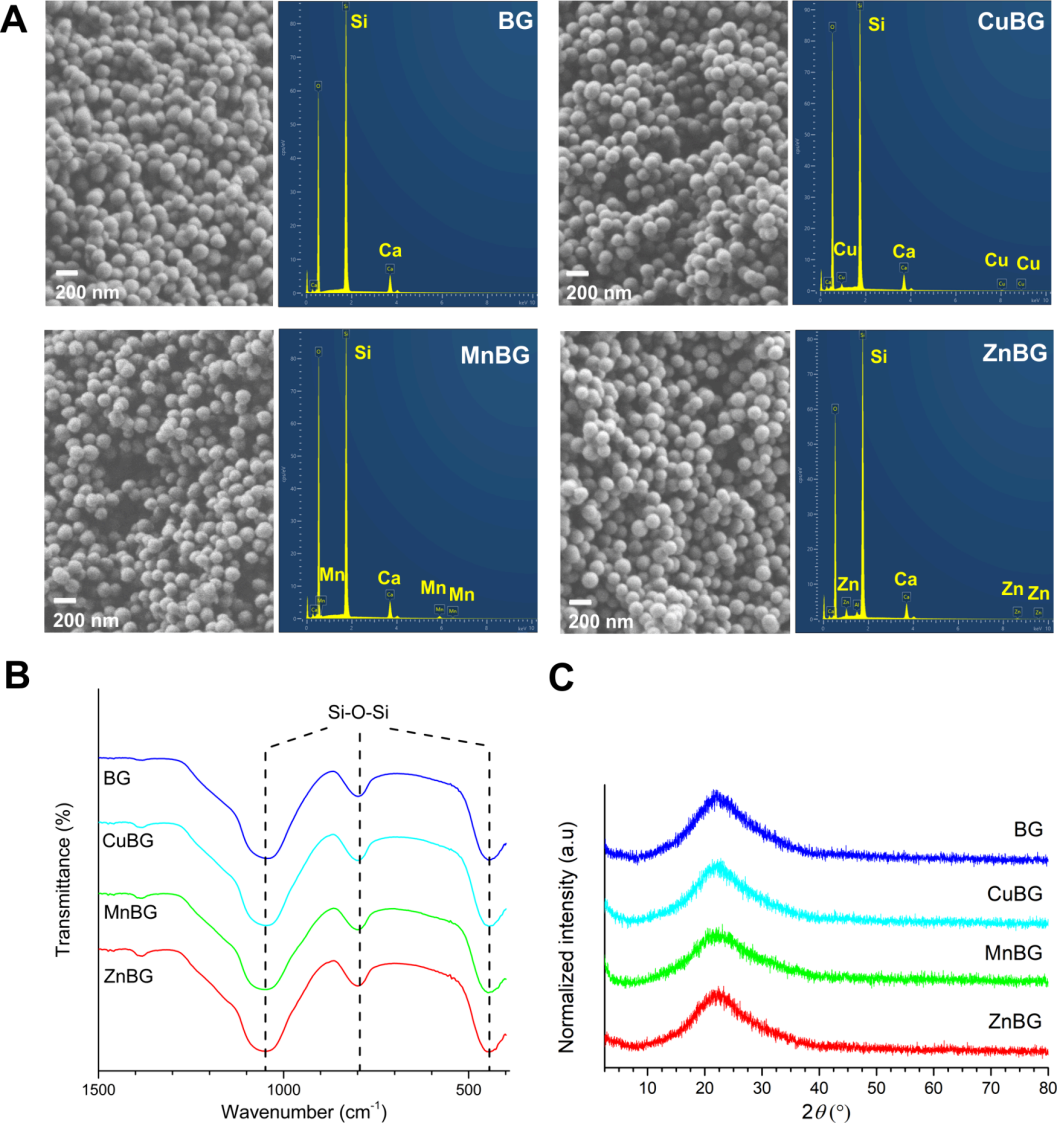
(A) SEM micrographs and EDX spectra, (B) FTIR spectra, and (C) XRD patterns of undoped and Cu-/Mn-/Zn-doped BG nanoparticles.

### 3.2 Size Distribution and Morphology of BG/Chitosan Microparticles

Prepared undoped and doped BG nanoparticles were used as bioactive fillers for chitosan-copper microparticles prepared by an electrohydrodynamic atomization process. Figure 2 shows the size distribution and sphericity factor of composite microparticles containing 10 wt % undoped and Cu-, Mn-, or Zn-doped BG nanoparticles. All samples possess narrow size distribution with an average particle size of 45 ± 6, 42 ± 6, 42 ± 7, and 43 ± 6 μm for BG/Cht, CuBG/Cht, MnBG/Cht, and ZnBG/Cht, respectively, indicating the similar sizes of all composite systems. Additionally, good sphericity indicated by an average value close to 1 was achieved by the EDHA process.

**Figure 2:**
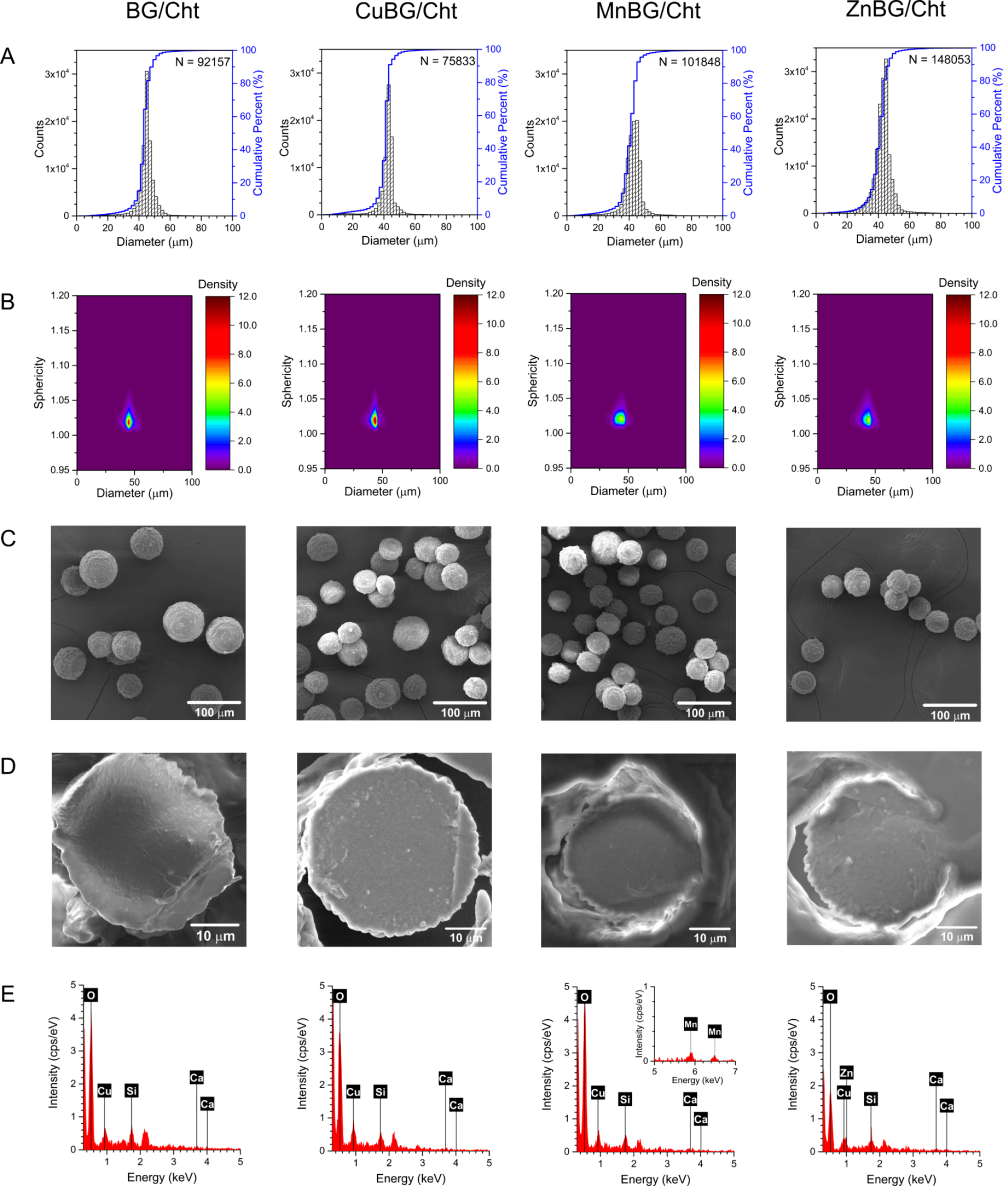
(A) Particle size distribution, (B) sphericity, and (C) morphology of composite microparticles containing 10 wt % of undoped BG (BG/Cht), Cu-doped BG (CuBG/Cht), Mn-doped BG (MnBG/Cht), and Zn-doped BG (ZnBG/Cht) nanoparticles. (D) Morphology and (E) elemental analysis of the composite microparticles in a cross-sectional view.

The morphology and elemental composition of the EDHA-produced microparticles were investigated by SEM/EDX (Figure 2). The surface of the microparticles is seen to be as rough and wrinkled, which could be due to the applied electric field and surface tension of the polymer solution when the drop is pulled away from the tip of the needle. The imaging of the cross-section of the microparticles revealed a bulk structure with no visible agglomerates of BG nanoparticles. The EDX spectra showed the presence of silicon and calcium as a confirmation of the presence of BG nanoparticles and copper originating from the chitosan matrix in all samples. Additionally, manganese and zinc were detected for MnBG/Cht and ZnBG/Cht microparticles, respectively. Higher intensity of the characteristic copper peak was observed in the CuBG/Cht sample as a result of the Cu presence in both the organic and inorganic phases.

### 3.3 Swelling Behavior and Ion Release

The water absorption of composite microparticles was investigated in two physiological media, PBS and complete cell culture medium (DMEM) after 24 h of immersion. As seen in Figure 3, the microparticles exhibit larger volumes, i.e., diameter, when in contact with an aqueous solution with respect to their dry state. BG/Cht, CuBG/Cht, and ZnBG/Cht microparticles preserved their bluish color after 24 h in PBS, which implies that copper ions involved in chitosan cross-link remained within the polymer matrix, while MnBG/Cht microparticles remained brown due to the color of Mn-doped BG nanoparticles. On the other hand, all composite microparticles became more transparent after 24 h of immersion in DMEM. The swelling capacity of composite microparticles was calculated by the wet-to-dry volume ratio. For BG/Cht, CuBG/Cht, MnBG/CHT, and ZnBG/Cht, the swelling capacity in DMEM was 2.55, 3.04, 2.77, and 2.86, while in PBS it was 2.43, 2.49, 2.25, and 2.29, respectively. Although a similar increment in particle diameter, ranging from 2.29 to 3.04, was observed in both media, the swelling capacity of all composites was higher in DMEM than in PBS medium.

**Figure 3:**
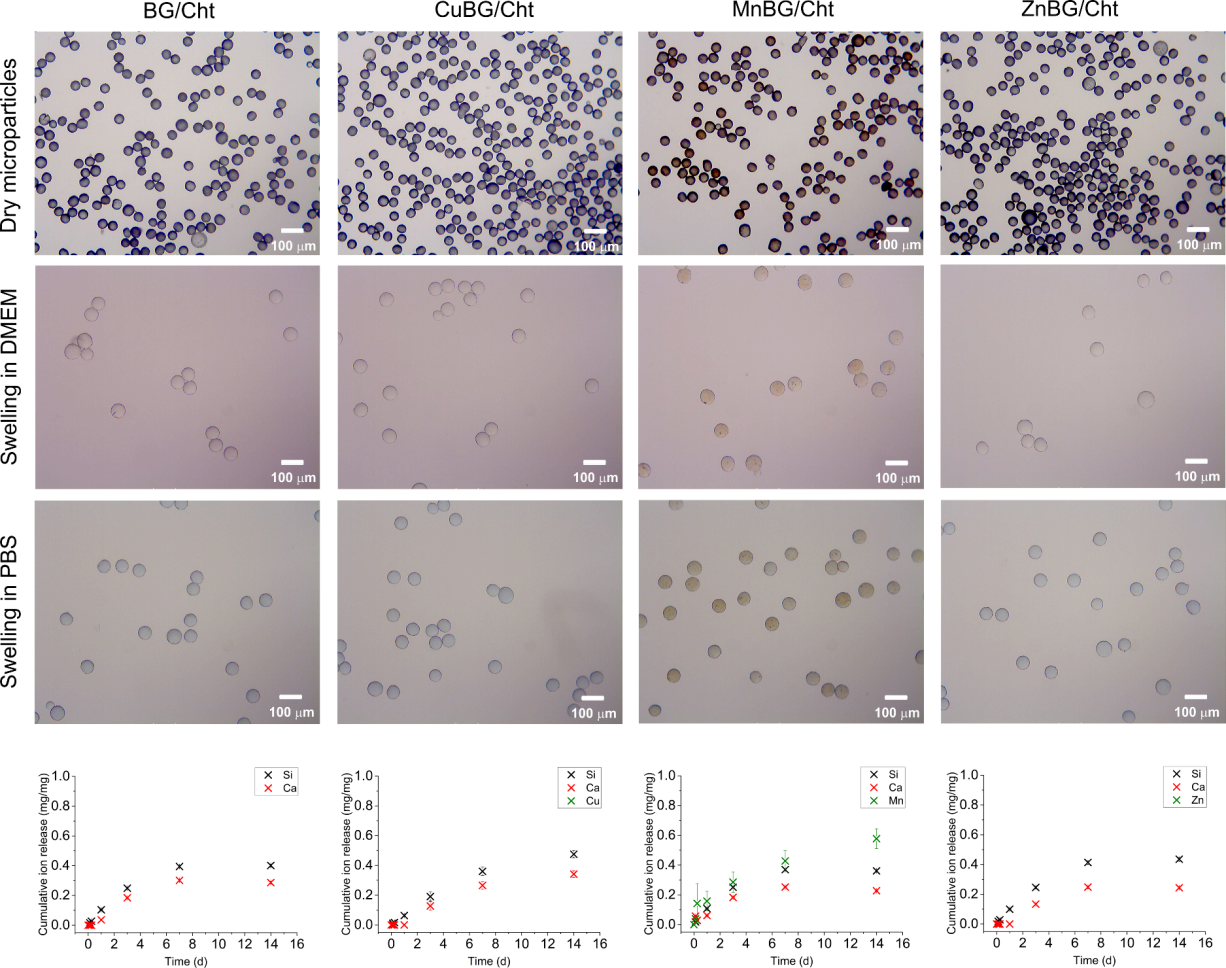
Optical micrographs of dry and swollen composite microparticles and results of the cumulative release (mass of released element per nominal composition of element) from composite microparticles during 14 days of incubation in PBS as measured by ICP-OES.

The ion release profiles of the composite microparticles were investigated at physiological pH in PBS for different incubation periods (Figure 3). Our previous works enlighten the ion release from similar undoped and doped MBGNs in more complex media such as cell culture medium^13,33,35^ and SBF.^36–38^ Here, we aimed to investigate the dissolution properties of MBGNs in PBS as a model of a simple ionic solution mimicking the pH of blood plasma, as we did previously.^39^ Microparticles with undoped BG showed an initial rapid release of Si ions followed by a slower liberation after 3 days. A similar Si release profile was detected for the MnBG/Cht and ZnBG/Cht microparticles. Calcium ions were detected after the first day of incubation, with a similar release trend for the BG-, MnBG-, and ZnBG-containing samples. The concentrations of released Si and Ca ions from the CuBG/Cht sample showed pronounced release in the first 7 days, followed by continuous release up to 14 days. Manganese ions were continuously released over 14 days from the MnBG/Cht microparticles. Interestingly, Cu and Zn ions were not detected throughout the entire release experiment. The EDX analysis confirmed the presence of copper in all composite microparticles, since it was used as a physical cross-linker for chitosan chains; however, the quantities measured by ICP-OES were below the detection limit.

### 3.4 Evaluation of Bioactivity in SBF

To assess the ability to induce the formation of a hydroxycarbonate apatite (HCA) layer, the microparticles were incubated in SBF at 37 °C for 21 days. After 7 days of incubation, all composite systems maintained similar surface morphology as nontreated samples, with no visible precipitates (Figure 4). More wrinkled surfaces were observed, while the spherical shape was preserved. Pronounced wrinkling effects could be a result of the different water absorption abilities of the polymer and filler during immersion in SBF and the subsequent drying step.

**Figure 4:**
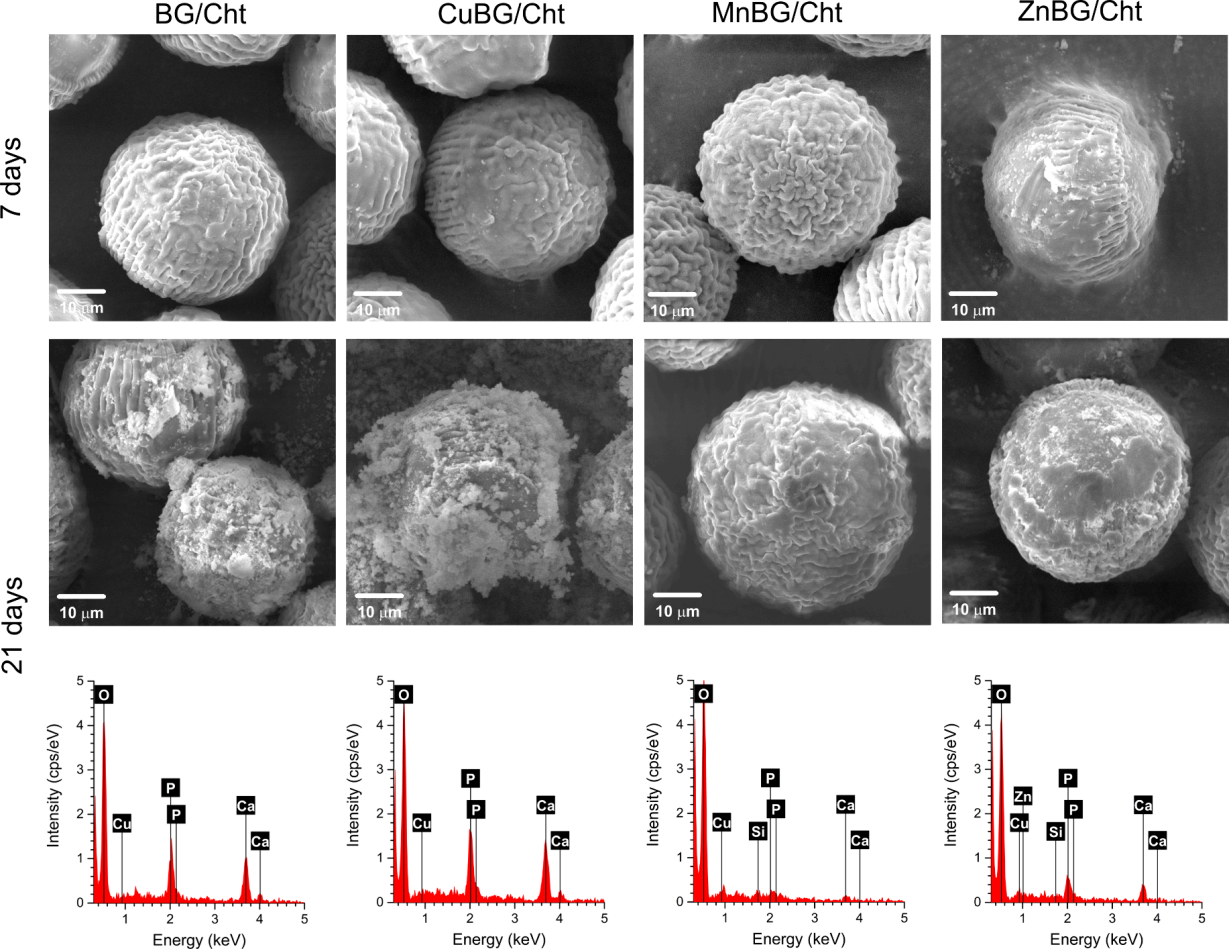
SEM micrographs and EDX spectra of composite microparticles containing 10 wt % of undoped BG (BG/Cht), Cu-doped BG (CuBG/Cht), Mn-doped BG (MnBG/Cht), or Zn-doped BG (ZnBG/Cht) during 21 days of incubation in SBF.

Visible precipitates on the microparticles’ surface were observed after 21 days of incubation for BG/Cht, CuBG/Cht, and ZnBG/Cht samples, corresponding to a calcium phosphate phase, as confirmed by EDX spectra. On the contrary, the MnBG/Cht sample does not exhibit the precipitated layer of calcium phosphate on its surface even after 21 days of incubation, while the surface morphology did not significantly alter.

### 3.5 Cytotoxicity and VEGF Expression

The influence of BG/Cht, CuBG/Cht, MnBG/Cht, and ZnBG/Cht microparticles on the cell viability was assessed by indirect and direct tests. An indirect cytotoxicity test was performed on MG-63 cells using microparticle extracts of different concentrations (0.01, 0.1, and 1 mg/mL) collected after 1 and 7 days of material soaking in DMEM (Figure 5). The viability of 70% with respect to the nontreated cells (Ctrl) was used as the cytotoxicity threshold (ISO Standard No. 10993-5:2009). The viability of MG-63 cells treated with 0.01 and 0.1 mg/mL of materials after 1 and 7 days of material soaking was comparable to the negative control or even exceeded it, indicating noncytotoxicity. At a concentration of 1 mg/mL, a slight decrease in cell viability was observed for all composite microparticles, while the 7-day extract of the ZnBG-containing sample was cytotoxic.

**Figure 5:**
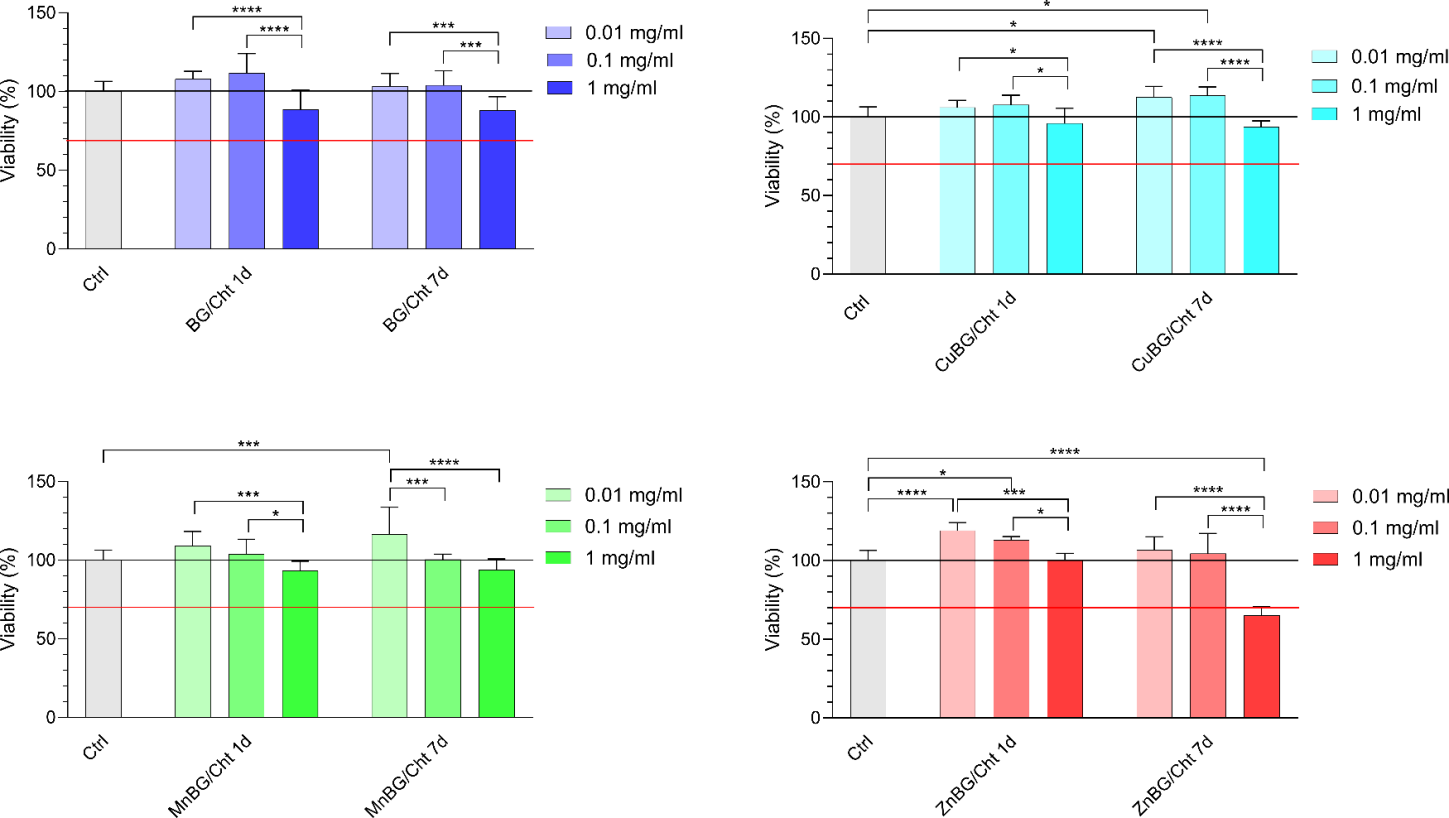
Indirect cytotoxicity assay on MG-63 cells after 24 h of treatment with extracts of BG/Cht, CuBG/Cht, MnBG/Cht, and ZnBG/Cht microparticles of different concentrations.

Direct cytotoxicity assays were performed on MG-63 and HDFa cells at microparticle concentrations of 0.1 and 0.5 mg/mL (Figure 6) after 24 and 72 h of incubation. The cell viability of MG-63 cells cultured with 0.1 mg/mL of microparticles was comparable to that of the nontreated cells after 24 and 72 h of culture, while 0.5 mg/mL of microparticles caused a decrease in cell viability to the cytotoxicity threshold after 24 h. The marginal cytotoxicity for MG-63 cells was maintained for the 0.5 mg/mL MnBG/Cht system after 72 h of culture, while the adaptation of the cells to the environment, indicated by higher cell viability, was observed for the rest of the composites. Compared with the cell viability obtained by the indirect tests, it is notable that the material in direct contact with the cells has a pronounced cytotoxic effect.

**Figure 6:**
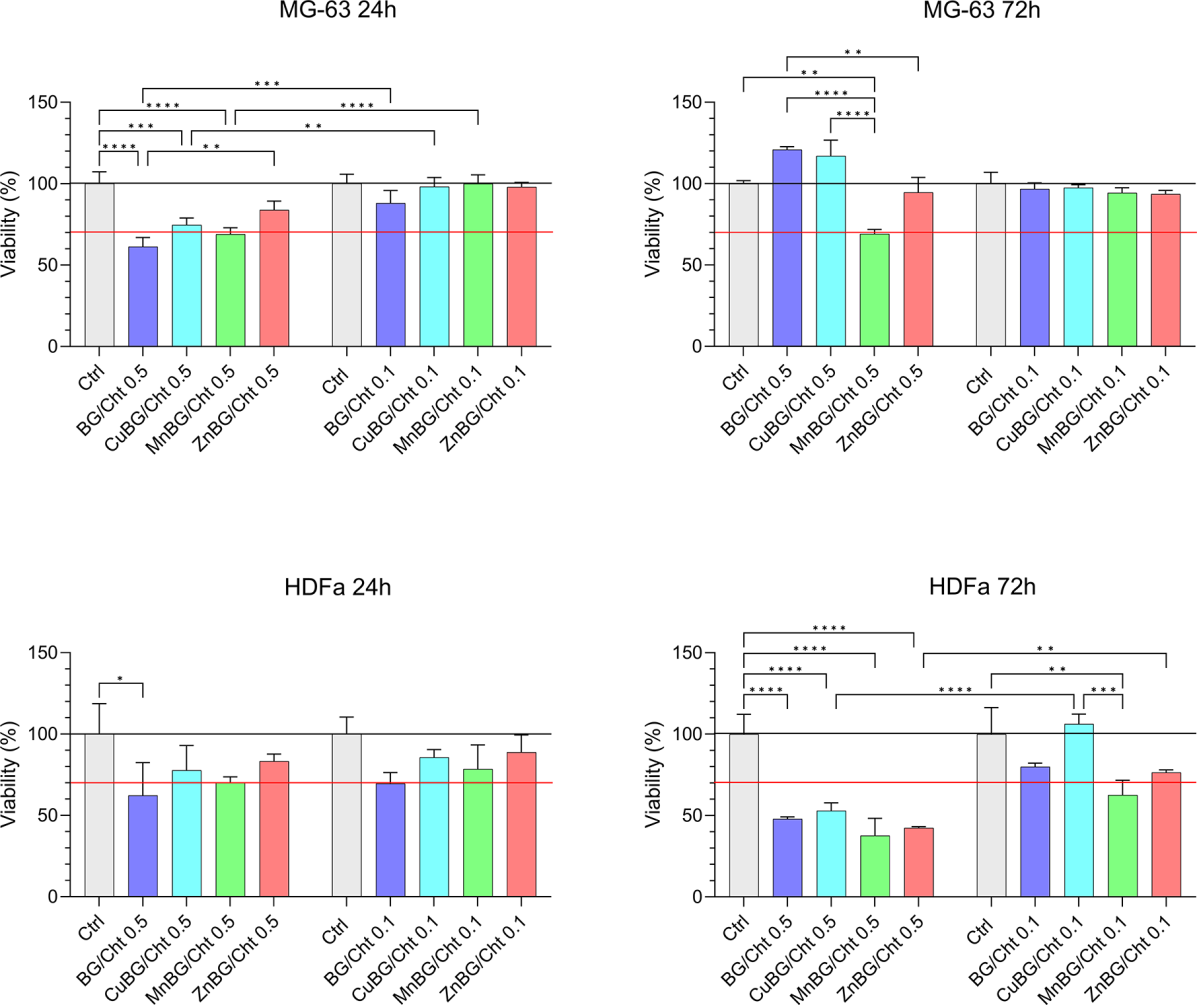
Direct cytotoxicity assay on MG-63 and HDFa cells during 72 h of culture with BG/Cht, CuBG/Cht, MnBG/Cht, and ZnBG/Cht at different concentrations (0.1 and 0.5 mg/mL).

HDFa cells cultured in direct contact with the microparticles at 0.1 and 0.5 mg/mL for 24 h exhibited a lower viability with respect to the negative control. However, with the exception of the BG/Cht sample at 0.5 mg/mL, no significant difference was observed. After 72 h, BG/Cht, MnBG/Cht, and ZnBG/Cht showed marginal cytotoxicity at 0.1 mg/mL, while CuBG/Cht was comparable to the nontreated cells, indicating no harmful effect of copper ions incorporated within the organic and inorganic phases. Direct contact of cells with materials at a higher concentration caused a significant decrease in cell viability below the cytotoxicity threshold for all systems after 72 h of culture.

The quantification of the VEGF expression by MG-63 and HDFa cells was performed using an ELISA assay after 72 h of cell culture in direct contact with the materials at concentrations of 0.1 and 0.5 mg/mL. VEGF levels are presented relative to the nontreated cells (Ctrl) and depicted in Figure 7. For MG-63 cells, the VEGF expression was similar to that of the control cells at a concentration of 0.1 mg/mL, indicating the absence of the microparticle’s impact on VEGF expression. However, at a concentration of 0.5 mg/mL, a significantly higher level of VEGF expression was induced by the MnBG/Cht composite.

**Figure 7:**
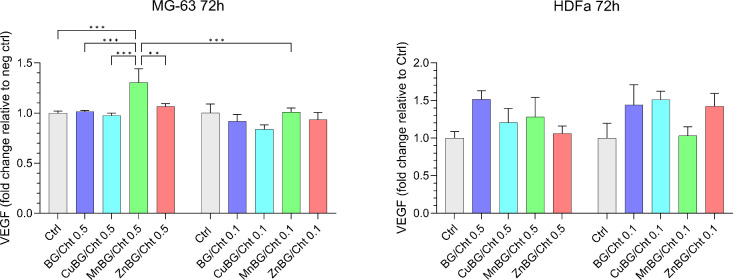
VEGF expression by MG-63 and HDFa cells after 72 h of culture with different concentrations of microparticles (0.1 and 0.5 mg/mL) determined by ELISA.

HDFa cells showed a slight increase in VEGF expression induced by the 0.1 and 0.5 mg/mL concentrations of the BG/Cht sample, while the lower concentrations of CuBG/Cht and ZnBG/Cht microparticles induced higher VEGF expressions with respect to the control cells. Even though the VEGF expression by HDFa cells cultured in contact with composite microparticles had a slight increase, there was no significant difference compared to the nontreated cells.

## 4 Discussion

Microparticles are mainly considered useful delivery carriers of drugs and biomolecules (e.g., growth factors or enzymes) introduced into scaffolds. When it comes to irregularly shaped bone defects or defects with a small entrance, monolithic scaffolds are difficult to implant. The injectability of microparticles makes them a suitable alternative for minimally invasive bone defect filling.^40^ Different synthetic and natural polymers have been selected for microparticle production depending on their application. Natural polymers have shown better biocompatibility with respect to synthetic ones. Among natural polymers, chitosan is shown to be a versatile polymer for functionalization and pH-responsive delivery. Chitosan-based microparticles and microcapsules have been utilized as delivery systems for lysozyme, BMP-2, alendronate, albumin, doxorubicin, etc.^41–45^ Besides, the formation of calcium phosphate on the surface of chitosan-based microparticles was also investigated in order to synthesize composite microparticulate delivery systems.^44,46^ However, very few studies have focused on the application of chitosan-bioceramic microparticles for injectable bone defect-filling materials.^47,48^

A growth factor-free approach for treating bone defects has been a hot topic in recent years. Biologically active ions, an alternative to growth factors, are emerging to impact biological activity to biomaterials.^11,13,33^ In this context, the usage of inorganic ions including strontium (Sr^2+^), magnesium (Mg^2+^), zinc (Zn^2+^), copper (Cu^2+^), silver (Ag^+^), and cobalt (Co^2+^) as stimulators for osteogenesis has been reported, simply added into solution or incorporated into bioceramics, bioactive glasses, or polymer matrices. In this work, we used BG nanoparticles and chitosan as carriers of therapeutic ions in the form of composite microparticles potentially used as individual or combined microparticulate systems with tailored biological properties. The incorporation of different metal ions into bioactive glasses of different compositions is a very active area of research, leading to bioactive formulations that induce specific cell responses.^49^ In a previous study, mesoporous BG nanoparticles doped with Cu^2+^, Mn^2+^, or Zn^2+^ ions showed the potential for local delivery of ions whose osteogenic properties were demonstrated on human marrow-derived mesenchymal stromal cells (BMSCs).^13^ Similar BG nanoparticles were prepared in this work with a slight change in the portions of oxide components, which did not significantly impact the size, shape, and morphology of the fabricated nanoparticles.

### 4.1 Production and Structure of Composite Microparticles

The EDHA process gave uniform composite microparticles with a narrow size distribution, making it a suitable production technique for chitosan microbeads. All composite systems possess comparable average sizes and distributions, suggesting a repeatable production process independently of the composition of the BG nanoparticles. Furthermore, the surface morphology of different composite microparticles remained rough and wrinkled, which could be a favorable surface topography for cell adhesion and proliferation due to increased contact area between cells and the material.^50^ In line with previous studies on chitosan-copper microparticles,^21,23^ the incorporation of metal ion-doped BG nanoparticles did not significantly influence the size and shape of chitosan microparticles. The spherical shape and narrow size distribution were preserved after immersion in PBS and DMEM, while microparticles were transformed into microgels by absorbing a large amount of water. The hydrogel nature of composite microparticles indicated by the high swelling capacity in physiological solution originates from a large number of amino and hydroxyl groups of chitosan, while copper ions were responsible for the good stability of the chitosan matrix. The slightly higher swelling capacity of all composite microparticles in DMEM compared to PBS medium can be attributed to the increased interaction between the amino groups of chitosan, copper ions and and the amino acids present in the complete cell culture medium.

### 4.2 Ion-Releasing Behavior and Acellular *in Vitro* Bioactivity

During swelling in PBS, bioactive ions are released from the composite microparticles with a significant release of silicon ions regardless of the dopant type present in the BG nanoparticles. The observed burst release of silicon ions is characteristic of BGs due to the dissolution, specifically for nanosized particles, followed by slower release at a longer incubation time. MnBG-containing microparticles showed a continuous release of Mn^2+^ ions, compared to the nondetectable concentration of respective dopant from CuBG- and ZnBG-containing samples, with a continuous release of calcium ions up to 7 days of incubation. Different release profiles may be connected to the different electronegativity, atomic radii, and coordination of network modifiers. Few theoretical models have been proposed to explain the ion release from biologically inactive glasses during corrosion.^51,52^ On the contrary, the ion release kinetics of bioactive glasses is more complex due to the concomitant precipitation of HCA on their surfaces.^53^ The proposed models could align with release profiles of ions from our MBGNs, meaning, the initial rapid release of Si reflects the dissolution of weakly constrained regions (e.g., nonbridging oxygen sites). The stochastic approach could be used to explain the diffusion and dissolution pathways of modifier sites occupied by the dopants. Still, the incorporation of MBGNs into the chitosan matrix further complicates the adapted application of the proposed models. On the contrary, the release of Cu ions for CuBG-containing microparticles and Zn ions from ZnBG/Cht microparticles was not detected by ICP-OES during the whole incubation period. In a previous study, the inspection of ion release in cell culture medium (DMEM) from similar Cu-, Zn-, or Mn-doped mesoporous BG formulations indicated the highest release of Cu^2+^ ions, followed by Zn^2+^ ions, while Mn^2+^ ions were released at the lowest concentration for the same doping level of SiO_2_–CaO based BGs, as a result of the strongest bonding within the glass structure.^13,33^ When in contact with water-based solutions, the surface of SiO_2_-based BGs is attacked by H^+^ or H_3_O^+^ ions, leading to a rapid exchange with network modifier ions (Ca^2+^ or Na^+^).^54^ The disruption of the glass network allows the release of silanol groups and the continuity of ionic exchange between the glass and the surrounding solution. Certainly, the composition of BGs, meaning the type of network modifier and portions of each oxide component, dictates the dissolution rate. It can be assumed that the initial dissolution of undoped and doped BG nanoparticles was induced by steps in microparticle preparation that involved the dispersion of BG nanoparticles into acidic chitosan solution and subsequent neutralization and washing of microgels in NaOH and water, respectively. Stronger bonding in Mn-doped BG could be responsible for the measurable release of Mn^2+^ ions from the chitosan microparticles during incubation in PBS.

Another interesting observation is the absence of copper ions that would be released from all of the composite systems. This behavior indicates the formation of a strong chelate between copper ions and chitosan functional groups, with good stability in ionic solutions such as PBS. On the contrary, such bonds would be easily disrupted by another molecule containing amino and hydroxyl groups. Transition metal ions such as Cu^2+^, Zn^2+^, and Mn^2+^ possess binding affinity toward different amino acid forming complexes.^55–57^ Among them, several amino acids are the main components of the cell culture medium that would affect the stability of the chitosan-copper chelate, which can be observed from microparticles after immersion in DMEM (Figure 3). Hence, the liberation of copper ions from the chitosan matrix is expected to be pronounced in the cell culture medium, influencing the cytotoxicity of such materials.

The acellular bioactivity test was performed primarily to detect the formation of CaP deposits on the surface of the microparticles as the initial indication of the bioactive character of the materials, e.g., the ability to form a CaP deposit on the surface and in this way to present an osteoconductive surface to the host bone in the intended application. According to SEM analysis, all composite microparticles did not show distinctive formation of the HCA layer within 7 days of incubation. After 21 days, the surface of BG/Cht and CuBG- and ZnBG-containing microparticles was covered with precipitates of the calcium phosphate phase, confirmed by EDX analysis. Our previous studies investigated the bioactivity of similar BG nanoparticles by analyzing CaP deposits during incubation in SBF and measuring the ion release.^36,37^ Studies showed that calcium ions were released from similar undoped and doped mesoporous BG nanoparticles, while P was consumed during CaP precipitation. The same behavior was expected from undoped and Cu-/Mn- and Zn-doped MBGNs used in this study. The released calcium ions from the BG nanoparticles, together with Ca^2+^ and PO_4_^3–^ ions from the solution, are involved in precipitation reactions that form the HCA layer over the silica gel formed during immersion.^54^ On the contrary, MnBG-containing microparticles did not show an inorganic layer on their surfaces. Characteristic calcium and phosphorus bands in the EDX spectra were not detected. The bioactivity of SiO_2_–CaO BG nanoparticles was maintained after the incorporation of Cu or Zn ions, which coincides with previous studies.^9,58–61^ Different formulations of Mn-doped BGs have also induced the formation of apatite layer after incubation in SBF during different periods;^62–65^ however, this was not observed for the prepared MnBG-containing composite microparticles after 21 days. In SiO_2_-based glasses, manganese ions act as network modifiers where calcium ions are partially substituted by Mn^2+^ ions. A continuous release of Mn^2+^ ions from microparticles, as indicated by an ion release study performed in PBS, is also expected in SBF, which could in turn impact the rate of HCA formation on the sample surface.^65^

### 4.3 Cell Biology Characterization with Indirect and Direct Cell Cultures

Cytotoxicity assays by sample extracts and in direct contact with the microparticles were conducted at varying material concentrations to explore the impact on cell viability. Indirect tests showed noncytotoxicity of the materials for all concentrations and incubation periods. The rapid dissolution of undoped and doped BG nanoparticles was expected to affect the viability of MG-63 cells; however, the precipitation of released ions in DMEM may diminish their influence on cell metabolism. In contrast, microparticles in direct contact with cells had a more pronounced cytotoxic effect. This can be partially attributed to the rough surface morphology of particles, which enables effective contact with cells and thus facilitates localized and faster ion exchange, amplifying their effect. MG-63 cell culture in direct contact with microparticles indicated slight cytotoxicity at higher concentrations after 24 h. A lower material concentration enabled viability comparable to that of the nontreated cells. The cytotoxicity was reduced after 3 days of culture for almost all composite microparticles, except for the MnBG/Cht system, where cell viability remained at the cytotoxicity limit. Manganese ions are involved in bone development and remodeling and aid in the collagen synthesis that provides structural support to bone.^66^ In addition to the therapeutic effect, Mn ions possess anticancer activity through rich redox chemistry, potentially inhibiting tumor growth,^67,68^ which could be a reason for the decreased viability of MG-63 cells in direct contact. On the other hand, HDFa cells showed greater sensitivity to all composite microparticles. This may be attributed to their nontumorigenic nature, making them more sensitive to oxidative imbalance, compared to stress-adapted cancer cells.^69^ All samples were cytotoxic after 24 and 72 h of direct contact with the cells at a concentration of 0.5 mg/mL. A marginal cytotoxic effect of BG/Cht and MnBG/Cht microparticles was also observed at lower material concentrations after 24 h of direct contact with HDFa cells. At this point, the presence of copper in all composite microparticles should be considered. The toxicity of copper was also shown to be dose dependent, leading to cell death at higher concentrations. Therefore, the possibility of a synergic effect of copper ions from the polymer matrix and dopant from the BG nanoparticles could exist in terms of decreasing the cell viability. On the contrary, the CuBG/Cht system showed comparable cell viability to nontreated HDFa cells after 3 days of culture at a concentration of 0.1 mg/mL. Nevertheless, the dissolution profile of the proposed composite microparticles should be explored in cell culture medium to clarify the relative effects of ions incorporated in the organic and inorganic phases of the microbeads.

### 4.4 Angiogenic Potential – VEGF Expression

The angiogenic potential of composite microparticles was explored by quantification of VEGF expressed by MG-63 and HDFa cells cultured in direct contact with the materials. VEGF has been shown to be one of the biomolecules critical in the earliest stages of vasculogenesis, as well as later in angiogenesis.^10,70^ When released, VEGF triggers the liberation, migration, and proliferation of endothelial cells and induces the formation of prevascular, tubular structures.^71^ The VEGF expression by MG-63 cells in contact with a lower material concentration was comparable to that of the nontreated cells. On the other hand, a significant increase in VEGF concentration was detected for cells in contact with the MnBG/Cht sample at higher concentration with respect to CuBG/Cht, ZnBG/Cht, and nontreated cells. The VEGF expression has also been associated with oxidative stress generated by reactive oxidative species (ROS) (such as hydrogen peroxide) as products of cell metabolism.^72,73^ According to the cytotoxicity assay performed on MG-63 cells, the MnBG/Cht sample (at a concentration of 0.5 mg/mL) caused a decrease in the cell viability after 72 h. Considering the redox activity of Mn^2+^ ions at high concentrations, a significantly higher expression of VEGF could be a result of oxidative stress.

When cultured with a higher quantity of microparticles, HDFa cells expressed slightly more VEGF or a concentration comparable to that of nontreated cells, which could also be associated with poor cell viability. Certainly, nontoxic concentrations of proposed microparticles need to be used to describe angiogenic stimulation. The viability of HDFa cells cultured with 0.1 mg/mL CuBG/Cht was the only one comparable to the nontreated cells, indicating no harmful effect (Figure 6). In parallel, the cells expressed slightly higher concentrations of VEGF with respect to the nontreated group, however, with the absence of significance. The angiogenic potential of copper ions has been broadly studied, as a medium supplement,^10,11,33,59,60,74^ incorporated into polymer matrices/hydrogels^75–77^ or BG/bioceramics.^25,78^ Copper ions are described to mimic hypoxic conditions by upregulating HIF-1α, which in turn upregulates angiogenic genes such as VEGF. Despite the stimulatory properties of copper, the right therapeutic window is still unknown. Nonetheless, the present findings provide directions for further exploration of the proposed composite systems as angiogenic materials.

### 4.5 Future Perspectives

The inspection of microparticle dissolution in cell culture medium with and without serum supplementation is needed to define the impact of therapeutic ions on cell behavior. In these composite systems, metal ions were incorporated into both the polymer matrix and the inorganic filler, which resulted in different release kinetics. Consequently, cell viability is affected by Cu^2+^ ions from the chitosan matrix and by Cu^2+^, Mn^2+^, or Zn^2+^ ions released from the MBGNs. Considering the same concentration of Cu^2+^ ions bonded to chitosan in all systems, it can be assumed that the main impact on cell viability was due to the metal ions released from the BG nanoparticles. However, a recent study^74^ showed that the combination of different ions, i.e., Cu^2+^ and Co^2+^ ions, decreased the viability of human umbilical vein endothelial cells (HUVECs) with respect to the usage of individual ions due to different roles of ions in ROS formation. Furthermore, the angiogenic response was enhanced when HUVECs were cultured individually compared to the culture with both Cu^2+^ and Co^2+^ ions. From this point of view, further studies on different cell types should include cell culture experiments using nontoxic concentrations of materials, which could enlighten the influence of each metal ion, and dissolution studies in cell culture medium to correlate ion release profiles with their biological properties.

## 5 Conclusions

The electrohydrodynamic atomization process successfully produced uniform, spherical chitosan-based composite microparticles with a narrow size distribution consistent across different doped mesoporous BG nanoparticles. Microparticles are stable microgels in water solutions, with slightly higher swelling in cell culture medium. Due to BG-nanoparticle dissolution, silicon ions were rapidly released and were accompanied by the continued release of calcium in all samples. Additionally, continuous release of manganese ions from MnBG/Cht was confirmed in PBS. All samples, except MnBG/Cht, showed bioactivity in SBF and formed a calcium phosphate layer. Most likely, continuous Mn^2+^ release is affecting mineralization.

Indirect assays on MG-63 cells showed that all materials were noncytotoxic at lower concentrations, while at 1 mg/mL and 7 days the extract of the ZnBG/Cht sample was cytotoxic. Cytotoxicity was more pronounced at higher concentrations and with prolonged treatment in direct contact, while MnBG/Cht was the most cytotoxic. Similarly, expression of VEGF was increased in MG-63 cells treated with a higher concentration of MnBG/Cht, probably due to oxidative stress from Mn^2+^ ions. HDFa cells expressed VEGF levels comparable to nontreated cells.

While the microparticles showed bioactivity, their ion release in the complete cell culture medium, cytotoxicity, and pro-angiogenic properties, especially to nontumorigenic cells, must be carefully studied. The prepared composite samples differ by dopant ion due to variation in mesoporous BG nanoparticles used. Further studies on ion dissolution kinetics are needed to better understand their biological effects.

## Data Availability

The data presented in this study are available on request from the corresponding authors.
